# How does area-level deprivation depress an individual’s self-rated health and life satisfaction? Evidence from a nationwide population-based survey in Japan

**DOI:** 10.1186/s12889-021-10578-2

**Published:** 2021-03-17

**Authors:** Takashi Oshio, Hiromi Kimura, Toshimi Nishizaki, Takashi Omori

**Affiliations:** 1 Institute of Economic Research, Hitotsubashi University, 2-1 Naka, Kunitachi, Tokyo, 186-8603 Japan; 2 Survey Research Center, 3-13-5 Nihonbashi, Chuo-ku, Tokyo, 103-0027 Japan; 3 Japan Cabinet Office, 1-6-1 Nagatacho, Chiyoda-ku, Tokyo, 100-8914 Japan; 4 Osaka University, 1-7 Machikaneyama Toyonaka, Osaka, 560-0043 Japan

**Keywords:** Area-level deprivation, Multilevel mediation analysis, Self-rated health, Subjective well-being

## Abstract

**Background:**

Area-level deprivation is well known to have an adverse impact on mortality, morbidity, or other specific health outcomes. This study examined how area-level deprivation may affect self-rated health (SRH) and life satisfaction (LS), an issue that is largely understudied.

**Methods:**

We used individual-level data obtained from a nationwide population-based internet survey conducted between 2019 and 2020, as well as municipality-level data obtained from a Japanese government database (*N* = 12,461 living in 366 municipalities). We developed multilevel regression models to explain an individual’s SRH and LS scores using four alternative measures of municipality-level deprivation, controlling for individual-level deprivation and covariates. We also examined how health behavior and interactions with others mediated the impact of area-level deprivation on SRH and LS.

**Results:**

Participants in highly deprived municipalities tended to report poorer SRH and lower LS. For example, when living in municipalities falling in the highest tertile of municipality-level deprivation as measured by the *z*-scoring method, SRH and LS scores worsened by a standard deviation of 0.05 (*p* < 0.05) when compared with those living in municipalities falling in the lowest tertile of deprivation. In addition, health behavior mediated between 17.6 and 33.1% of the impact of municipality-level deprivation on SRH and LS, depending on model specifications.

**Conclusion:**

Results showed that area-level deprivation modestly decreased an individual’s general health conditions and subjective well-being, underscoring the need for public health policies to improve area-level socioeconomic conditions.

## Background

It is well known that area-level socioeconomic conditions have a contextual association with an individual’s health outcomes [1–3]. Many studies have indicated that area-level deprivation, which reflects various aspects of an area’s socioeconomic positions, can affect the health outcomes of its residents, including mortality [4–7], morbidity [8–11], mental health [12, 13], poor birth outcomes [9, 14, 15], and health risk behavior [16, 17]. The impact of area-level deprivation on health matters for not only public health policies but also macroeconomic and social policies committed to income redistribution, labor market regulations, and other issues related to social welfare.

To capture small-area socioeconomic deprivation, a variety of deprivation measures have been developed [18, 19]. These measures generally cover multiple dimensions of deprivation, such as income, employment, education, social class, and housing conditions. To construct a single index of deprivation, the most straightforward way is to sum standardized scores (*z*-scores) of each dimension with an equal weight (e.g., Townsend and Carstairs indices [20, 21]) or different weights (e.g., Jarman index [22]). To avoid normative judgment that is inevitably involved in any weighting, the principal component and factor analysis approaches, which assign a set of weights that statistically best explain the variation in the data, have also been often used [23–25]. Because all of these approaches are known to have both advantages and disadvantages and have no clear theoretical background [18], it may be useful to compare their results and assess their robustness.

Regarding the association between area-level deprivation and health, two issues remain to be addressed. First, the impact on self-rated health (SRH), which represents overall health conditions [26, 27], or subjective well-being (SWB), which is often expressed by life satisfaction (LS), has been relatively understudied, compared to the impact of area-level deprivation on mortality, morbidity, or other specific health outcomes [4–16]. Meanwhile, many studies have shown that individual-level SWB measures were related to area-level objective well-being. Residents in areas with more favorable socio-economic characteristics were found to be happier and more satisfied with life [28–31]. Studies have also examined the association between area-level income inequality or poverty and an individual’s SRH or SWB [32–34]. Hence, it is of interest to know whether the same is true for area-level deprivation, which captures other domains of area-level socioeconomic positions rather than just income.

Second, the factors that may mediate the impact of area-level deprivation on SRH and LS need to be further addressed. Specific factors such as the availability of alcohol, the physical environment, and maternal health have been found to mediate the impact of neighborhood deprivation on alcohol consumption, some health biomarkers, and preterm birth, respectively [15, 35, 36]. In the case of the impact on SRH and SWB, health behavior and/or interactions with neighbors/friends, both of which are well known to affect them [37–40], could be potential mediators. In particular, the mediating effect of health behavior, if any, would have an important policy implication because public policies for healthy lifestyle promotion could then be expected to mitigate the adverse impact of area-level deprivation.

Keeping these issues in mind, this study conducted a multilevel analysis to examine how an individual’s SRH and LS were associated with municipality-level deprivation in Japan, where regional disparities in health resumed a widening trend in the mid-1990s [41, 42]. An increasing number of studies have examined the importance of area-level socioeconomic conditions as a contextual determinant of health among Japanese people. Specifically, some studies examined how municipality-level socioeconomic positions are associated with mortality or life expectancy [4], while other studies investigated how neighborhood deprivation is related to all-cause mortality [6], as well as the risk of incidence, mortality, and survival from cancers [11]. However, more investigation into its impact on SRH or LS as well as its potential mediators is needed for a more comprehensive understanding of the relevance of area-level deprivation for an individual’s well-being.

## Methods

### Study sample

This study used data obtained from a population-based, nationwide internet survey conducted as a research project of the Cabinet Office (CAO) of the Japanese government in October 2019 and again in February 2020. The survey was conducted in accordance with Japan’s Statistics Law, which governs the statistical, legal, ethical, and other rules for surveys conducted by the government. Informed consent was obtained from all participants. We obtained the data of the survey with the permission of the CAO; therefore, ethics approval was not required for the current study.

We distributed the questionnaires to the registrants of an internet survey company. We planned to collect data from approximately 15,000 participants: around 10,000 from the survey in 2019 and the remaining 5000 from the survey in 2020. We divided the targeted sample into two groups. First, we distributed 11,280 registrants equally between each of the 47 prefectures, between men and women, and among five age groups (aged 15–24, 25–34, 35–44, 45–59, and 60+). Thus, each prefecture-gender-age group consisted of 24 individuals. Next, we allocated the remaining 4245 registrants to each gender-age group in each prefecture in proportion to each prefecture’s actual population size. When we closed the survey, we had obtained data from 15,574 participants. It should be noted that this construction of the dataset made the residents living in the metropolitan areas underweighted compared to the actual population.

We used municipality, which is the basic unit of local administration in Japan, for a unit of area. From a total of 1741, the current study collected data from 1273 municipalities with the number of participants ranging from 1 to 257 (mean [M] 12.2 and standard deviation [SD] 25.4). Excluding data from municipalities with less than ten participants and also participant data missing key variables, we used data from 12,461 participants (6157 men and 6304 women) living in 366 municipalities, where the number of participants from each municipality ranged between 1 and 257 (M 34.0 and SD 38.5).

### Measures

#### Individual-level variables

Regarding SRH, the survey asked the participants to answer the question: “How do you feel about your health condition?” by choosing good, somewhat good, average, somewhat poor, or poor. We constructed a continuous variable of SRH by allocating 1 to poor and 5 to good (the higher, the better). The survey also asked the participants to answer the question: “In general, how satisfied are you with your life?” by responding on an 11-point scale (0 = not satisfied at all to 10 = highly satisfied). We constructed a continuous variable of LS (the higher, the more satisfied).

We also considered health behavior and interactions with others. Regarding health behavior, the survey asked the participants whether they were usually doing the following for their health: 1) eating a balanced diet, 2) exercising, 3) getting enough sleep, 4) refraining from smoking, 5) refraining from excess alcohol consumption, 6) avoiding the accumulation of stress, 7) going for regular checkups, 8) doing nothing in particular, and 9) other. We constructed a continuous variable of health behavior by adding up the number of chosen items from 1 to 7. As for interactions with others, the survey asked participants how often they interacted with friends or others. We constructed a continuous variable of interactions with others by allocating 7, 7/2, 1, 2.5/4, 1/4, 1/16, 1/48, and 0 to almost every day, three or four times a week, once a week, twice or thrice a month, sometimes a year, once a year, and no one to interact with, respectively.

We considered individual-level deprivation in terms of income, education (graduated from junior high school only), and job status (unemployed). Regarding income, we adjusted the reported amount by household size by dividing household income by the square root of the number of household members. We subsequently defined income poverty as an adjusted household that fell below the official poverty line, which is 1.22 million JPY at 2015 price, as defined by the Japanese Ministry of Health, Labor and Welfare (MHLW) [43]. As individual-level covariates, we considered gender, age group (29 or below, 30s, 40s, 50s, and 60 or above), marital status (having a spouse or not), and survey years (2019 or 2020).

#### Municipality-level variables

At the municipality level, we selected seven indicators: 1) the percentage of unemployed persons, 2) the percentage of persons who had an educational attainment of college or above, 3) taxable income per capita, 4) the percentage of owned houses, 5) the percentage of households with floor space per capita that was below the minimum level (defined by the Ministry of Land, Infrastructure, Transport and Tourism [44]); 6) the percentage of single-parent households, and 7) the percentage of aged single households. The choice of indicators largely followed those of preceding studies [4, 20–22]. Specifically, the indicators 1) to 4) correspond to general socioeconomic conditions, 5) represents the extent of household overcrowding, and 6) and 7) represent prevalence of vulnerable groups. We downloaded this data from the website database for municipality-level socioeconomic indicators, which are based on government surveys conducted around the year 2015. This official database is provided by the Ministry of Internal Affairs and Communications [45].

### Analytic strategy

We computed the municipality-level deprivation indices in two ways. First, we conducted the *z*-scoring method by summing the *z*-scores of each indicator. In this calculation, we revered the signs of taxable income per capita and the percentage of owned houses, both of which were expected to make a negative contribution to municipality-level deprivation. Second, we conducted a principal component analysis (PCA) and selected components for which the eigenvalue of the correlation matrix was more than one as significant dimensions. Two or three principal components were expected to be obtained as significant dimensions. We calculated scores based on each of these components as well as their sum as deprivation indices. We categorized each deprivation index into tertiles: low, moderate, and high.

For the statistical analysis, we estimated multilevel linear regression models to explain SRH/LS scores by binary variables of moderate and high levels of each deprivation index (using low deprivation as a reference) along with individual-level deprivation and covariates. We compared the results across different methods of constructing the deprivation index.

We then conducted a multilevel mediation analysis to examine whether and to what extent health behavior and interactions with others mediated the association between municipality-level deprivation and SRH/LS. In the case of health behavior for SRH, we estimated a structural equation model consisting of 1) a model to explain health behavior by deprivation, 2) a model to explain interactions with others by deprivation, and 3) a model to explain SRH by deprivation, health behavior, and interactions with others, and then calculated the mediation effects of health behavior and interactions with others based on the estimated parameters. Denoting the scores of health behavior, interactions with others, and SRH as *HBEHAV*, *INTERACT*, and *SRH*, respectively, and the binary variables of moderate and high deprivation as *Moderate* and *High,* respectively, we estimated the structural equation model for individual *j* living in municipality *i*:

$$ {HBEHAV}_{ij}={\beta}_{11}{Moderare}_i+{\beta}_{12}{High}_i+\left(\mathrm{individual}-\mathrm{level}\ \mathrm{variables}\right)+{u}_{1i}+{\varepsilon}_{1 ij},{INTERACT}_{ij}={\beta}_{21}{Moderate}_i+{\beta}_{22}{High}_i+\left(\mathrm{individual}-\mathrm{level}\ \mathrm{variables}\right)+{u}_{2i}+{\varepsilon}_{2 ij},{SRH}_{ij}={\beta}_{31}{Moderate}_i+{\beta}_{32}{High}_i+{\gamma}_1{HBEHAV}_{ij}+{\gamma}_2\ {INTERACT}_{ij}+\left(\mathrm{individual}-\mathrm{level}\ \mathrm{variables}\right)+{u}_{3i}+{\varepsilon}_{3 ij}, $$

where *u* denotes municipality-level fixed effects, and *ε* an error.

Based on the estimated regression coefficients, we calculated the mediating effects. For the impact of living in municipalities with high deprivation on SRH, we derived the mediating effects of health behavior and interactions with others as *β*_12_*γ*_1_ and *β*_22_*γ*_2_, respectively, using participants living in the municipalities. If the mediating effect was found significant, we further computed its proportion out of the entire impact of living in highly deprived municipalities on SRH as *β*_12_*γ*_1_/(*β*_32_ + *β*_12_*γ*_1_ + *β*_22_*γ*_2_) × 100% in the case of health behavior. Similarly, we computed the mediating effects of living in moderately deprived municipalities. We also repeated the same estimation procedure for LS. We used the software package Stata (Release 16) for the statistical analysis. Finally, we repeated a similar analysis by replacing the variable of overall health behavior with a binary variable of each of seven types of health behavior and examined their relative importance for SRH and LS. For all statistical analyses, we used the software package Stata (Release 15).

## Results

**Fig. 1:**
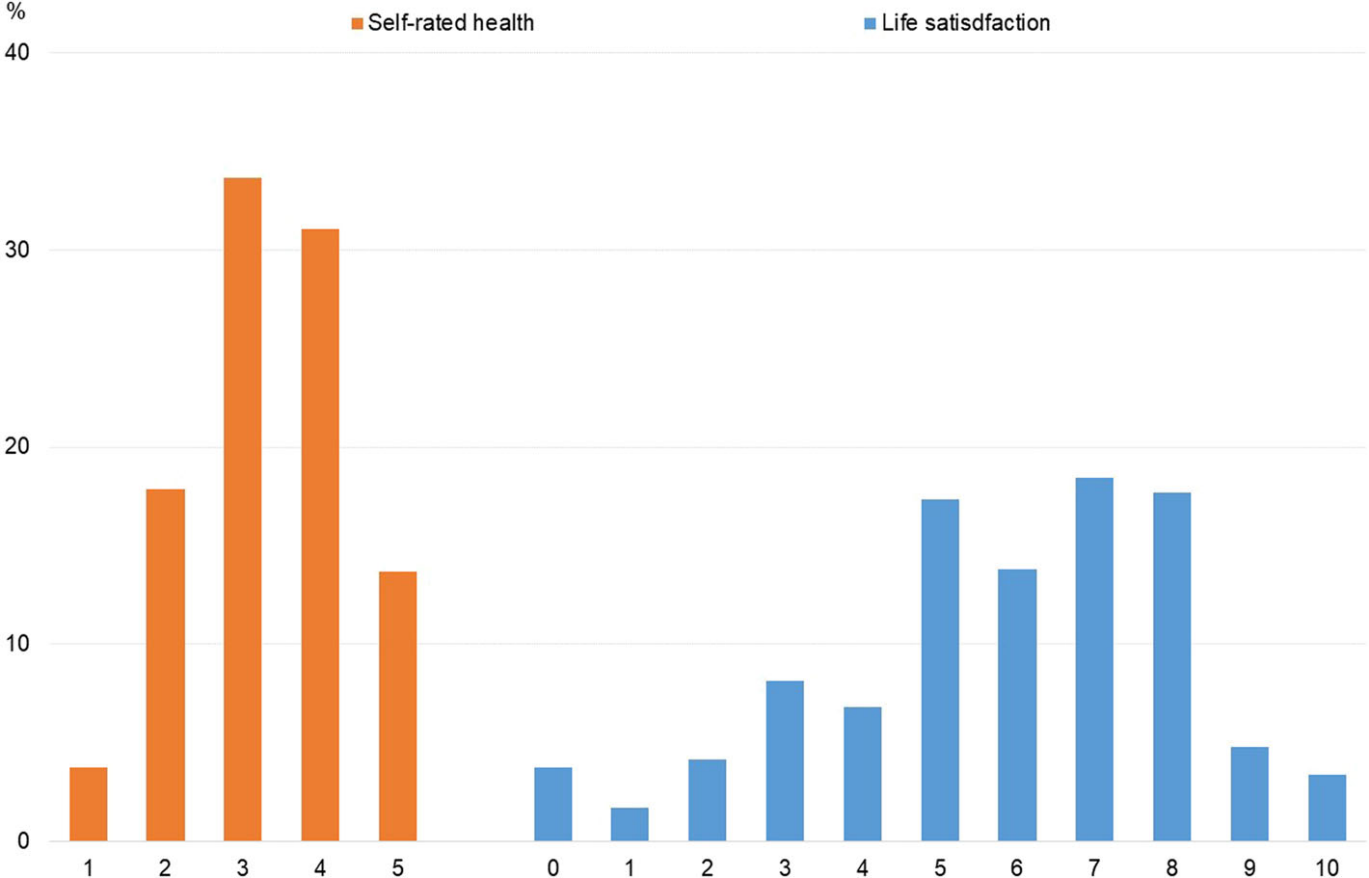
Distributions of self-rated health and life satisfaction (*N* = 12,461)

Table 1 summarizes the key features of the participants used in this study. No substantial differences were observed between samples collected in 2019 and 2020. In the entire sample, 12.9% of the participants had an income below the poverty line, 2.7% had not graduated from high school or above, 3.2% were unemployed, and 25.8% had no spouse. As is often the case of Internet surveys, the educational attainment of the respondents was biased upward; the proportion of those who had graduated from college or above was 45.5%, much higher than 26.6% in the MHLW’s Comprehensive Survey of Living Conditions in 2016 [41]. The SRH score had a M of 2.7, a SD of 1.0, and the LS score had an M of 4.2, and a SD of 2.3. Figure 1 presents the histograms of SRH and LS. While SRH had a central, single peak at 3, LS had two peaks at 5 and 7 and somewhat more left-skewed distribution. These two scores were highly correlated with each other, with a correlation coefficient being 0.38 (*p* < 0.001).

**Table 1:** Key features of the study sample

	All		Surveyed in 2019		Surveyed in 2020	
Proportion (%)						
Men	49.4		49.5		49.3	
Women	50.6		50.5		50.7	
Income poverty	12.9		12.7		13.3	
Educational attainment						
Junior high school	2.7		2.8		2.6	
High school	51.7		51.6		51.9	
College or above	45.5		45.6		45.5	
Unemployed	3.2		3.4		2.7	
Having no spouse	25.8		26.1		25.3	
	*M*	*SD*	*M*	*SD*	*M*	*SD*
Income^a^	3.95	(6.52)	3.96	(6.75)	3.94	(6.06)
Age	43.9	(16.3)	43.7	(16.3)	44.2	(16.3)
Self-rated health (1–5)^b^	2.7	(1.0)	2.7	(1.0)	2.7	(1.0)
Life satisfaction (0–10)^c^	4.2	(2.3)	4.2	(2.4)	4.2	(2.3)
*N*	12,461		8246		4215	

^a^ Household size adjusted. Annual, million JPY

^b^ The higher, the poorer

^c^ The higher, the less satisfied

Table 2 presents pairwise correlation coefficients across municipality-level SRH, LS, and the seven deprivation indicators. As seen in this table, taxable income was positively associated with both SRH and LS scores, and the percentage of persons with the highest educational attainment was positively associated with SRH scores. Other indicators had no significant or only modest associations with SRH or LS scores. Meanwhile, the seven deprivation indicators had relatively high correlations with each other.

**Table 2:** Pairwise correlation coefficients across municipality-level self-rated health, life satisfaction, and deprivations (*N* = 366)

	1	2	3	4	5	6	7	8	*M*	*SD*
1. Self-rated health ^a^									3.32	(0.25)
2. Life satisfaction ^b^	0.43^***^								5.79	(0.53)
3. Taxable income per capita	0.13^✝^	0.15^**^							1.44	(0.44)
4. Unemployment rate	–0.09^✝^	0.10^✝^	−0.35^***^						6.4	
5. % of persons who graduated from college or above	0.10^✝^	0.22^***^	0.72^***^	− 0.26^***^					16.6	
6. % of aged single households	− 0.02	0.06	− 0.56^***^	0.56^***^	− 0.54^***^				10.7	
7. % of single-parent households	−0.10^✝^	0.08	−0.23^***^	0.25^***^	− 0.26^***^	0.19^***^			1.7	
8. % of owned houses	−0.11^*^	−0.10^✝^	− 0.48^***^	−0.15^**^	− 0.44^***^	0.05	0.06		65.4	
9. % of houses below minimum floor space	0.07	0.06	0.48^***^	0.07	0.45^***^	−0.24^***^	−0.06	− 0.87^***^	5.4	

^a^ Range: 1–5 (the higher, the better)

^b^ Range: 0–10 (the higher, the more satisfied)

^***^ *p* < 0.001, ^**^ *p* < 0.01, ^*^ *p* < 0.05^, ✝^ *p* < 0.1

Table 3 shows the results of the principal component analysis. We obtained the first and second principal components, which, when combined, accounted for 78.8% of the total variance in the data. The first component had highly positive loadings on single-parent households, unemployment, and aged single households, and highly negative loadings on taxable income and highest educational attainment. The second component had highly positive loadings on diminished floor space, unemployment, and aged single households, and a negative loading on house ownership. Judging from the proportion of the variance accounted for by a set of selected seven indicators and the directions of loadings on them, we can roughly argue for the validity of their choice as indicators of municipality-level deprivation. We subsequently constructed three deprivation indices, corresponding to the first and second principal components and their combinations. As mentioned above, we categorized these indices into tertiles.

**Table 3:** Results of principal component analysis on municipality-level deprivation

Indicator	First component	Second component
Taxable income per capita	−0.51	0.13
% of unemployed persons	0.38	0.36
% of persons who graduated from college or above	−0.49	0.09
% of aged single households	0.30	0.32
% of single-parent households	0.49	−0.07
% of owned houses	0.03	−0.60
% of houses below minimum floor space	−0.12	0.61

Table 4 presents the estimation results of multilevel linear regression models to explain SRH scores by municipality-level deprivation derived by the *z*-scoring method and individual-level variables. The SRH score was standardized by its sample M and SD. The table compares the estimation results across Model 1 (which included individual-level deprivation only), Model 2 (which included municipality-level deprivation only), and Model 3 (which included both). As seen in the table, the estimated coefficients on deprivation variables at both levels were largely the same across three models, suggesting that deprivation variables at two levels were associated with SRH largely independently from each other. We confirmed similar results for other indices of municipality-level deprivation and also for LS (not reported but available upon request). These results suggest that it may be largely reasonable to treat individual-level deprivation variables as control variables and focus on the extent of which health behavior and interactions with others mediated the impact of municipality-level deprivation on SRH and LS. Equally important, Table 3 confirms that moderate and high municipality-level deprivation worsened SRH scores by 0.05 SD (standard error [SE]: 0.02 SD), compared to low deprivation. The table also shows that the magnitude of the association between individual-level deprivation (in terms of income, education, and employment status) and SRH was in the range of 0.21–0.27 SD, much higher than that for municipality-level deprivation.

**Table 4:** Estimation results of a multilevel regression model to explain self-rated health Dependent variable: self-rated health (standardized)^a^

	Model 1		Model 2		Model 3	
	Coef.	SE	Coef.	SE	Coef.	SE
Municipality-level deprivation						
Moderate			−0.06^*^	(0.02)	−0.05^*^	(0.02)
High			−0.05^*^	(0.02)	−0.05^*^	(0.02)
Individual-level						
Income poverty	0.21^***^	(0.03)			−0.21^***^	(0.03)
Lowest educational attainment	−0.22^***^	(0.05)			−0.22^***^	(0.05)
Unemployed	−0.27^***^	(0.05)			−0.27^***^	(0.05)
Having no spouse	−0.15^***^	(0.02)	−0.17^***^	(0.02)	−0.15^***^	(0.02)
Female	0.02	(0.02)	0.01	(0.02)	0.02	(0.02)
Age: 30–39 years	−0.19^***^	(0.03)	−0.17^***^	(0.03)	−0.19^***^	(0.03)
Age: 40–69 years	−0.40^***^	(0.03)	−0.39^***^	(0.03)	−0.40^***^	(0.03)
Age: 50–59 years	−0.53^***^	(0.03)	−0.52^***^	(0.03)	−0.53^***^	(0.03)
Age: 60 or above	−0.41^***^	(0.03)	−0.39^***^	(0.03)	−0.41^***^	(0.03)
Surveyed in 2020	0.01	(0.02)	0.01	(0.02)	0.01	(0.02)

*N* = 12,461 (in 366 municipalities)

^a^ The higher, the poorer

^***^ *p* < 0.001, ^*^ *p* < 0.05

Table 5 compares the estimated impact of municipality-level deprivation on SRH and LS across eight model specifications. The most noticeable finding is that high municipality-level deprivation worsened SRH and LS scores by 0.05–0.06 SD in all model specifications except for the PCA method using the second component (bottom). The results for moderate deprivation were somewhat more mixed; its impact was even larger than high deprivation for LS in some cases while it did not have a significant impact on others. We also found that the second principal component had no association with SRH or LS.

**Table 5:** Estimated association of municipality-level deprivation with self-rated satisfaction and life satisfaction: multilevel regression models^a^

Dependent variable	Self-rated heath^b^		Life satisfaction^c^	
Municipality-level deprivation	Coef.	*SE*	Coef.	*SE*
*z*-scoring method				
Moderate	− 0.05^*^	(0.02)	− 0.06^**^	(0.02)
High	−0.05^*^	(0.02)	−0.05^*^	(0.02)
PCA^d^ method				
First and second components				
Moderate	−0.04	(0.02)	−0.09^***^	(0.02)
High	−0.05^*^	(0.02)	−0.06^**^	(0.02)
First component				
Moderate	−0.01	(0.02)	−0.00	(0.02)
High	−0.06^**^	(0.02)	−0.05^*^	(0.02)
Second component				
Moderate	0.01	(0.03)	−0.03	(0.03)
High	0.01	(0.02)	−0.02	(0.03)

*N* = 12,461 (in 366 municipalities)

^a^ Controlled for individual-level deprivation and covariates (not reported)

^b^ The higher, the better. Standardized

^c^ The higher, the more satisfied. Standardized. ^d^ Principal component analysis

^***^ *p* < 0.001, ^**^ *p* < 0.01, ^*^ *p* < 0.05

Table 6 summarizes the mediation analysis for SRH using the *z*-scoring method. We found that health behavior was negatively associated with high municipality-level deprivation (top panel), while interactions with others had no significant relationship with deprivation (second). We also found that both health behavior and interactions with others were negatively associated with poor SRH (third). Hence, we can assume that there may be only one mediation process: health behavior mediated the impact of high deprivation. Indeed, Table 6 shows that health behavior mediated 21.1% (SE: 9.5%) of the association between high deprivation and SRH, while there was no other significant mediation effect.

**Table 6:** Mediating effects of health behavior and interactions with others on the impact of municipality-level deprivation (derived by the *z*-scoring method) on self-rated health^a^

		Coef.	SE
Health behavior^b^			
Moderate deprivation	*β*_11_	−0.04	(0.02)
High deprivation	*β*_12_	−0.05^*^	(0.02)
Interactions with others^b^			
Moderate deprivation	*β*_21_	−0.03	(0.02)
High deprivation	*β*_22_	−0.03	(0.02)
Self-rated health^b, c^			
Moderate deprivation	*β*_31_	−0.04^*^	(0.02)
High deprivation	*β*_32_	−0.04	(0.02)
Health behavior	*γ*_1_	0.21^***^	(0.01)
Interactions with others	*γ*_2_	0.09^***^	(0.01)
Mediating effect on the impact of high deprivation on self-rated health			
Health behavior	*β*_12_*γ*_1_	−0.011^*^	(0.005)
% proportion: *β*_12_*γ*_1_/(*β*_32_ + *β*_12_*γ*_1_ + *β*_22_*γ*_2_) × 100		21.1^*^	(9.5)

*N* = 12,461 (in 366 municipalities)

^a^ Controlled for individual-level covariates (not reported)

^b^ Standardized

^c^ The higher, the better

^***^ *p* < 0.001, ^*^ *p* < 0.05

We obtained similar results for other combinations of the outcome (SRH or LS) and methods (*z*-scoring and PCA [using the combined components or the first component]), although we have not presented them to conserve space. In all cases, we found that (1) health behavior was negatively associated with high deprivation, (2) interactions with others were not associated with deprivation, and (3) both health behavior and interactions with others were negatively associated with outcome scores, as seen in Table 6.

These results allowed us to concentrate on the mediating effect of health behavior on the impact of high deprivation. Table 7 compares these effects across six combinations of outcomes (SRH and LS) and methods (*z*-scoring and PCA methods [using the combined components or the first component]). Table 7 shows that in all cases, the impact of high deprivation was mediated by health behavior, as already seen in the case of the combination of SRH and the *z*-scoring method. The proportional impact of high deprivation on SRH by health behavior ranged from 20.3% (SE: 10.1%) to 25.2% (SE: 10.0%). For LS, the proportion was in a somewhat wider range, from 17.6% (SE: 8.7%) to 33.1% (SE: 13.8%).

**Table 7:** Mediating effect of health behavior on the impact of high deprivation at the municipality level^a^

		Self-rated health^b, c^		Life satisfaction^b, d^	
			(SE)		(SE)
*z*-scoring method	Effect	−0.011^*^	(0.005)	−0.01^*^	(0.006)
	% proportion	21.1^*^	(9.5)	24.8^*^	(12.5)
PCA^e^ method					
First and second components	Effect	−0.01^*^	(0.00)	−0.01^*^	(0.01)
	% proportion	20.3^*^	(10.1)	17.6^*^	(8.7)
First component	Effect	−0.02^*^	(0.01)	−0.02^***^	(0.01)
	% proportion	25.2^*^	(10.0)	33.1^*^	(13.8)

*N* = 12,461 (in 366 municipalities)

^a^ Controlled for individual-level covariates (not reported)

^b^ Standardized

^c^ The higher, the better

^d^ The higher, the more satisfied

^e^ Principal component analysis

^***^ *p* < 0.001, ^*^ *p* < 0.05

Table 8 summarizes the estimation results for each of seven types of health behavior. We estimated the structural equation model separately for each behavior by replacing the variable of overall health variable by a binary variable of that behavior. For each health behavior, the table presents (1) the association with SRH and LS, (2) the association with high municipality-level deprivation (derived by the *z*-scoring method), and (3) the mediating effect of high deprivation on SRH and LH. While all seven types of health behavior were associated with SRH and LS, eating a balanced diet and getting enough sleep were most closely related to both of them. Meanwhile, the mediating effect of high deprivation was observed only for exercising and getting enough sleep.

**Table 8:** Estimation results for each health behavior^a^

	Self-rated health^b, c^		Life satisfaction^b, d^	
		(SE)		(SE)
Association with self-rated health (*γ*_1_)				
Eating a balanced diet	0.40^***^	(0.02)	0.39^***^	(0.02)
Exercising	0.36^***^	(0.02)	0.27^***^	(0.02)
Getting enough sleep	0.32^***^	(0.02)	0.38^***^	(0.02)
Refraining from smoking	0.13^***^	(0.02)	0.15^***^	(0.02)
Refraining from excess alcohol consumption	0.11^***^	(0.02)	0.18^***^	(0.02)
Avoiding the accumulation of stress	0.27^***^	(0.02)	0.40^***^	(0.02)
Going for regular checkups	0.08^***^	(0.02)	0.26^***^	(0.02)
Association with high deprivation^e^ (*β*_12_)				
Eating a balanced diet	−0.02	(0.01)	−0.02	(0.01)
Exercising	−0.03^**^	(0.01)	−0.03^**^	(0.01)
Getting enough sleep	−0.03^**^	(0.01)	−0.03^**^	(0.01)
Refraining from smoking	−0.00	(0.01)	−0.00	(0.01)
Refraining from excess alcohol consumption	0.00	(0.01)	0.00	(0.01)
Avoiding the accumulation of stress	−0.01	(0.01)	−0.01	(0.01)
Going for regular checkups	−0.01	(0.01)	−0.01	(0.01)
Moderating effect on the impact of high deprivation (*β*_12_*γ*_1_)				
Eating a balanced diet	−0.008	(0.005)	−0.007	(0.004)
Exercising	−0.011^**^	(0.004)	−0.008^**^	(0.003)
Getting enough sleep	−0.009^**^	(0.004)	−0.010^**^	(0.004)
Refraining from smoking	0.000	(0.001)	0.000	(0.002)
Refraining from excess alcohol consumption	0.000	(0.001)	0.001	(0.002)
Avoiding the accumulation of stress	−0.003	(0.003)	− 0.004	(0.004)
Going for regular checkups	−0.001	(0.001)	−0.002	(0.003)

^a^ Controlled for individual-level covariates (not reported)

^b^ Standardized

^c^ The higher, the better

^d^ The higher, the more satisfied

^e^ Derived by the *z*-scoring method

^***^ *p* < 0.001, ^**^ *p* < 0.01

## Discussion

The current study showed that municipality-level deprivation depressed and individual’s general health conditions, which were measured by SRH as well as SWB, which was represented by LS. The results did not differ significantly between the *z*-scoring and PCA methods. These findings were consistent with those obtained in previous studies and provided evidence of the contextual, adverse impact of area-level deprivation on health outcomes [4–17].

We obtained two additional observations. First, the impact of municipality-level deprivation was generally modest. Specifically, living in municipalities with moderate or high deprivation worsened SRH or LS scores by a 0.05 − 0.09 SD, compared to those living in municipalities with low deprivation, as observed in Table 5. The magnitude of this impact was one fifth to one fourth of that of the association of SRH with individual-level deprivation in terms of income (0.21SD), education (0.22SD), or employment status (0.27SD), as observed in Table 4. These results indicated that the impact of area-level deprivation on SRH or LS was in general much limited compared to individual-level deprivation.

Second, health behavior mediated the impact of high deprivation at the municipality level on SRH and LS. Interactions with others, which had a favorable association with SRH and LS, albeit to a lesser extent than health behavior, were not much affected by municipality-level deprivation. By comparison, area-level deprivation was found to discourage individuals from exhibiting healthy behavior. One possible explanation is that individuals living in highly deprived municipalities may have more chances to observe and be influenced by the unhealthy behavior of neighbors with low socioeconomic status.

Regarding the mediating effect of health behavior, three factors should be noted. First, the mediating effect worked only if area-level deprivation was high. This result suggests that area-level deprivation must be sufficiently high to make neighbors’ unhealthy behavior so commonly observed to affect an individual’s behavior. Second, the proportional impact of deprivation on SRH and LS mediated by health behavior ranged from 17.6 to 33.1%, meaning that health behavior is not a dominant, albeit non-negligible, mediator of the impact of area-level deprivation. Third, the results suggest that exercising and getting enough sleep exhibit greater mediating effects than other types of health behavior.

However, we cannot rule out the possibility that there may be important mediators other than health behavior and interactions with others. Indeed, studies have shown that perceptions of neighborhood and built environment mediated the impact of area-level deprivation on health outcomes [46, 47]. Even so, the results of the current study point to the possibility that policy measures to promote a healthy lifestyle can help to alleviate the adverse impact of area-level deprivation.

The current study has several limitations. First, the results may depend on the choice of measures of municipality-level deprivation. The selection of municipality-level indicators in this study was generally in line with preceding studies [4, 21–23] and we believe that they are able to capture a comprehensive picture of the socioeconomic positions of each municipality. However, we cannot rule out arbitrariness in the choice of indicators, and single indices of area-level deprivation cannot be free from criticism. We compared the results between *z*-scoring and PCA methods to examine the robustness of the estimated results. However, equal weighting in the *z*-scoring method requires more justification rather than simplicity or replicability, whereas the weights assigned to the indicators in the PCA method have no clear theoretical basis. In this regard, Nakaya et al.’s approach [6, 11, 48–50], which provides a foundation to the choice and weighting of area-level indicators by linking them to the individual-level poverty, must be a promising direction to develop better area-level deprivation indices.

Second, we should be cautious in interpreting the results of our mediation analysis. The implicitly assumed causations from municipality-level deprivation to health behavior and from health behavior to SRH and LS had no rigorous theoretical ground, and the temporal precedence of municipality-level deprivation over other variables was not guaranteed. Moreover, the observed mediating mechanism may have been confounded by a third variable.

Third, the study sample was not fully representative of the entire population in Japan, especially in terms of underweighted residents in the metropolitan areas. The observed results were not adjusted by weighted estimates taking into account the actual demographic distribution, probably causing estimation biases.

In addition to these key limitations, we recognize that cross-sectional analysis in the study cannot precisely identify causation from area-level deprivation to individual-level health and well-being. We also ignored the determinants of individuals’ choice of where to live and a possible time lag between deprivation and health outcomes. To address these issues, we would need a longitudinal dataset.

## Conclusions

This study showed that area-level deprivation modestly depresses an individual’s general health conditions and SWB, independent of individual-level deprivation. We also observed that health behavior mediated the impact of area-level deprivation. The results underscore the need for public health policies to improve area-level socioeconomic conditions and to promote healthy lifestyles to alleviate the negative impact of area-level deprivation.

## Data Availability

The data that support the findings of this study are available from the CAO, but restrictions apply to the availability of this data, which were used under admission for the current study, and so are not publicly available. Data are, however, available from the authors upon reasonable request and with permission from the CAO.

## Abbreviations

CAO: Cabinet office
LS: Life satisfaction
MHLW: Ministry of Health, Labour and Welfare
M: Mean
PCA: Principal component analysis
SD: Standard deviation
SE: Standard error
SRH: Self-rated health
SWB: Subjective well-being
